# Role of ETS2 gene in inflammatory bowel disease: A narrative review

**DOI:** 10.1097/MD.0000000000044234

**Published:** 2025-09-05

**Authors:** F.N.U. Aakash, Sunny Kumar, Aasta Kumari, F.N.U. Shweta, F.N.U. Rumela, F.N.U. Partab, Shivam Singla, Abida Parveen

**Affiliations:** a Department of Medicine, Ibn e Seena Hospital, Kabul, Afghanistan.

**Keywords:** colorectal cancer, ETS2, fibrosis, immune regulation, inflammatory bowel disease

## Abstract

The ETS2 gene, a member of the ETS (E26 transformation-specific) family of transcription factors, plays a critical role in the regulation of immune responses, epithelial barrier integrity, and fibrosis, all of which are central to the pathogenesis of inflammatory bowel disease (IBD). This review explores the molecular characteristics of ETS2, its involvement in immune dysregulation, and its contribution to IBD-associated complications, including fibrosis and colorectal cancer. ETS2 regulates key inflammatory pathways such as NF-κB and JAK-STAT, influencing cytokine production and immune cell polarization. Additionally, it affects epithelial barrier function by modulating tight junction proteins, thereby impacting intestinal permeability. Dysregulation of ETS2 expression can exacerbate intestinal inflammation, promote fibrosis, and increase the risk of colorectal cancer in IBD patients. Genetic variants of ETS2 have been associated with disease susceptibility, suggesting its potential as a biomarker for disease progression. Furthermore, targeting ETS2 may provide novel therapeutic strategies for IBD by modulating inflammatory pathways, restoring epithelial integrity, and preventing fibrosis and cancer. Understanding the role of ETS2 in IBD pathogenesis could lead to more personalized treatment approaches and improved clinical outcomes. This review highlights the potential of ETS2 as a therapeutic target and underscores the need for further research to elucidate its precise molecular mechanisms in IBD.

## 1. Introduction

Inflammatory bowel disease (IBD) is a chronic inflammatory condition of the gastrointestinal tract, primarily encompassing Crohn disease and ulcerative colitis.^[1]^ It is characterized by periods of remission and relapse, with symptoms such as abdominal pain, diarrhea, weight loss, and fatigue.^[2]^ The exact cause of IBD remains unclear, but it is widely recognized as a multifactorial disease involving genetic predisposition, environmental triggers, immune system dysfunction, and gut microbiota alterations.^[3]^ Over the years, the incidence of IBD has been increasing globally, particularly in industrialized nations, emphasizing the need for a deeper understanding of its underlying mechanisms to improve treatment strategies.^[4]^

Genetics plays a crucial role in the pathogenesis of IBD, as evidenced by familial clustering and genome-wide association studies (GWAS) as shown in Table 1.^[5]^ More than 200 genetic loci have been associated with IBD susceptibility, with many of them linked to immune regulation, epithelial barrier integrity, and microbial interactions.^[6]^ Notable genes such as NOD2, IL23R, and ATG16L1 have been extensively studied for their contributions to disease onset and progression.^[7]^ Among the emerging genetic factors, transcriptional regulators have gained significant attention due to their role in modulating inflammatory responses and cellular homeostasis.^[8]^ One such gene is ETS2, which has been increasingly recognized for its involvement in immune regulation and inflammation.^[9]^

**Table 1:** Genes involved in IBD.

Gene	Location	Role in IBD pathogenesis	Associated function/path way	Variants/polymo
ETS2	Chromosome 21q22.3	Regulates immune responses, epithelial barrier function, and fibrosis. Implicated in inflammation, epithelial integrity, and fibrosis.	NF-κB, JAK-STAT, TGF-β pathways, tight junction regulation	Variants associated with immune dysregulation and disease susceptibility
NOD2	Chromosome 16q12.1	Involved in the recognition of bacterial pathogens and regulation of innate immunity.	Innate immune response, autophagy	Variants associated with Crohn disease susceptibility
IL23R	Chromosome 1p31.3	Plays a key role in regulating the balance of T cell responses, particularly Th17 cells.	Th17 differentiation, cytokine production	Variants linked to both Crohn disease and ulcerative colitis
ATG16L1	Chromosome 2q37.1	Important for autophagy, a process that removes damaged cells and pathogens, affecting immune regulation and inflammation.	Autophagy, innate immune response	Variants linked to Crohn disease
TNF	Chromosome 6p21.3	A pro-inflammator y cytokine that plays a central role in immune response and inflammation in IBD.	Inflammatory cytokine signaling, immune cell activation	TNF gene polymorphisms linked to IBD and response to TNF inhibitors
IL-10	Chromosome 1q32.1	Anti-inflammato ry cytokine that regulates immune responses and prevents excessive inflammation.	Immune regulation, T cell differentiation	Variants associated with susceptibility to ulcerative colitis
JAK2	Chromosome 9p24.1	Involved in the JAK-STAT signalling pathway, which regulates immune responses and cell proliferation.	Cytokine signaling, immune cell differentiation	Polymorphisms linked to increased risk of IBD and other autoimmune disorders
TGF-β1	Chromosome 19q13.2	A key regulator of inflammation and fibrosis, modulating immune responses and tissue repair in the gut.	Fibrosis, immune regulation, wound healing	Variants associated with fibrotic complications in IBD
CARD15 (also NOD2)	Chromosome 16q12.1	Contributes to immune response by recognizing bacterial pathogens and influencing gut microbiome interactions.	Innate immunity, autophagy	Mutations associated with Crohn disease susceptibility

ETS = E26 transformation-specific, IBD = inflammatory bowel disease, JAK = janus kinase, TGF-β = transforming growth factor-beta.

ETS2, or ETS Proto-Oncogene 2, is a member of the ETS (E26 transformation-specific) family of transcription factors that regulate various cellular functions, including proliferation, differentiation, and apoptosis.^[10]^ Located on chromosome 21q22.3, ETS2 is known to influence immune responses, particularly through its role in macrophage activation, cytokine production, and epithelial cell function.^[11]^ Recent studies suggest that ETS2 may contribute to IBD pathogenesis by modulating key inflammatory pathways such as NF-κB and JAK-STAT signaling, both of which are central to the chronic inflammation observed in IBD.^[12]^ Given its potential role in immune dysregulation and epithelial barrier dysfunction, understanding the functional implications of ETS2 in IBD could provide novel insights into disease mechanisms and therapeutic targets.^[13]^

This review explores the role of the ETS2 gene in the pathogenesis of IBD, focusing on its involvement in immune regulation, epithelial barrier function, and potential therapeutic implications.

## 2. ETS2 gene: structure and function

The ETS2 gene, located on chromosome 21q22.3, encodes a transcription factor that belongs to the ETS family.^[14]^ This family is characterized by a conserved ETS DNA-binding domain, which allows these proteins to regulate the expression of various target genes involved in cell proliferation, differentiation, apoptosis, and immune responses.^[15]^ ETS2 shares structural similarities with other ETS family members, including an N-terminal pointed (PNT) domain and a C-terminal transactivation domain, both of which contribute to its ability to interact with co-regulatory proteins and modulate gene transcription.^[16]^ The expression of ETS2 is tightly controlled at both the transcriptional and posttranslational levels, ensuring precise regulation of its target genes in different cellular contexts.^[17]^

ETS2 plays a crucial role in cellular signaling, particularly in pathways associated with immune regulation and inflammatory responses.^[9]^ It functions as a transcriptional regulator of key cytokines and immune mediators, including TNF-α, IL-1β, and IL-6, which are known to drive inflammation in various disease states, including IBD.^[18]^ One of the primary mechanisms through which ETS2 exerts its effects is the activation of the NF-κB signaling pathway, a key regulator of inflammation and immune responses.^[9]^ By modulating NF-κB activity, ETS2 influences the expression of genes involved in leukocyte recruitment, cytokine production, and the maintenance of epithelial barrier integrity.^[9]^

Beyond its role in inflammation, ETS2 has been implicated in macrophage activation and polarization, critical processes in the pathogenesis of IBD.^[19]^ Studies have shown that ETS2 contributes to the differentiation of pro-inflammatory M1 macrophages, which release cytokines that exacerbate intestinal inflammation.^[20]^ Given its involvement in multiple inflammatory pathways, ETS2 is a potential therapeutic intervention target, particularly in conditions where immune dysregulation plays a central role.^[21,22]^ Understanding its precise function in immune homeostasis and intestinal epithelial integrity may provide novel insights into the pathogenesis and treatment of IBD.

ETS2 plays a significant role in immune dysregulation in IBD by influencing key inflammatory pathways and immune cell function.^[23–25]^

The influence of ETS2 extends to both the innate and adaptive immune responses.^[26]^ In the innate immune system, ETS2 is involved in the activation and polarization of macrophages, particularly promoting the pro-inflammatory M1 macrophage phenotype.^[27]^ These macrophages produce high levels of cytokines that exacerbate tissue damage in IBD.^[28]^ ETS2 also regulates dendritic cell maturation, affecting their ability to present antigens and activate T cells. In the adaptive immune response, ETS2 is linked to the differentiation of T helper cells, particularly Th1 and Th17 subsets, which are known to be key drivers of intestinal inflammation in IBD.^[29]^ The overactivation of these immune pathways leads to a persistent inflammatory state, contributing to mucosal injury and disease progression.^[30]^

Crosstalk between ETS2 and cytokines further amplifies immune dysregulation in IBD.^[31]^ ETS2 regulates the expression of IL-23, a cytokine that promotes Th17 cell expansion, thereby sustaining chronic inflammation.^[32]^ Additionally, it influences IL-10 signaling, which is critical for regulatory T cell function and immune homeostasis.^[33]^ Dysregulation of these interactions disrupts the balance between pro-inflammatory and anti-inflammatory responses, worsening disease severity.^[34]^ By orchestrating complex immune signaling networks, ETS2 emerges as a key factor in the immune dysregulation seen in IBD, making it a potential therapeutic target for modulating inflammation and restoring immune balance.^[35]^

## 3. ETS2 and epithelial barrier function

ETS2 plays a critical role in maintaining intestinal epithelial integrity, a key factor in the pathogenesis of IBD.^[36]^ The intestinal epithelium serves as the first line of defense, forming a selective barrier that prevents harmful pathogens and antigens from invading the underlying mucosa while allowing the absorption of nutrients.^[37]^ In IBD, epithelial barrier dysfunction contributes to increased intestinal permeability, leading to heightened immune activation and chronic inflammation.^[38]^ ETS2 has been implicated in the regulation of epithelial cell survival, differentiation, and repair processes, making it an important factor in mucosal homeostasis.^[39]^ Studies suggest that ETS2 influences epithelial regeneration by modulating the expression of genes involved in cell proliferation and apoptosis.^[40]^ Dysregulation of ETS2 expression can lead to impaired epithelial renewal, thereby compromising the structural integrity of the gut lining and exacerbating inflammation.^[41]^

One of the key mechanisms through which ETS2 affects epithelial barrier function is its regulation of tight junction proteins, which are essential for maintaining intercellular adhesion and controlling paracellular permeability.^[42]^ Tight junction proteins such as claudins, occludin, and zonula occludens proteins create a dynamic barrier that prevents the uncontrolled passage of luminal contents into the submucosa.^[43]^ ETS2 has been shown to modulate the expression of these proteins, with evidence suggesting that its dysregulation leads to tight junction disruption.^[44]^ Under inflammatory conditions, ETS2 expression may be altered in response to cytokines such as TNF-α and IFN-γ, which are known to induce tight junction disassembly.^[45]^ This results in a compromised epithelial barrier, allowing increased translocation of bacterial products and triggering a vicious cycle of immune activation and inflammation.^[46]^

Furthermore, ETS2 has been implicated in the regulation of epithelial-mesenchymal transition (EMT), a process in which epithelial cells lose their polarity and acquire mesenchymal characteristics, contributing to fibrosis and stricture formation in chronic IBD.^[47]^ By modulating key signaling pathways such as TGF-β and Wnt, ETS2 may influence the balance between epithelial repair and fibrosis, affecting disease outcomes.^[48]^ Given its multifaceted role in epithelial integrity, ETS2 represents a potential target for therapeutic interventions aimed at restoring barrier function and reducing intestinal permeability in IBD patients.^[49]^

## 4. Genetic variants of ETS2 and IBD susceptibility

### 4.1. Genome-wide association studies findings

GWAS have significantly advanced the understanding of genetic contributions to IBD, identifying multiple loci associated with disease susceptibility.^[50]^ While early GWAS efforts focused on well-established genes such as NOD2, IL23R, and ATG16L1, more recent studies have highlighted the role of transcription factors, including ETS2, in immune regulation and epithelial barrier function.^[51]^ The ETS2 gene, located on chromosome 21q22.3, has been implicated in immune-mediated diseases, with some variants showing potential associations with IBD.^[20]^ Although ETS2 has not been among the most frequently reported genes in GWAS for IBD, emerging data suggest that certain polymorphisms within or near ETS2 may influence disease susceptibility by modulating immune signaling and inflammatory pathways.^[52]^

Several GWAS analyses have identified single nucleotide polymorphisms (SNPs) in the ETS2 locus that correlate with altered immune responses and increased risk of chronic inflammatory conditions.^[20]^ These findings suggest that ETS2 may contribute to IBD pathogenesis by regulating pro-inflammatory cytokine expression and epithelial barrier integrity. However, further studies are needed to confirm these associations and clarify the precise mechanisms through which ETS2 variants impact IBD susceptibility and progression.

### 4.2. Functional Implications of ETS2 Polymorphisms in IBD

Genetic polymorphisms in ETS2 can have profound effects on its transcriptional activity, protein stability, and interaction with other signaling molecules.^[53]^ Variants leading to increased ETS2 expression may enhance inflammatory signaling by upregulating cytokines such as TNF-α, IL-1β, and IL-6, which are central to IBD pathophysiology.^[9]^ This overactivation can result in excessive immune cell recruitment, chronic inflammation, and tissue damage within the intestinal mucosa. Conversely, loss-of-function mutations or reduced ETS2 expression may impair the ability of epithelial cells to respond effectively to injury, compromising barrier integrity and promoting increased permeability to luminal antigens.^[54]^

Another key aspect of ETS2 polymorphisms is their potential influence on macrophage function and polarization. Certain ETS2 variants may promote a pro-inflammatory M1 macrophage phenotype, leading to persistent immune activation and cytokine production.^[9,19]^ This is particularly relevant in IBD, where dysregulated macrophage responses contribute to chronic inflammation and mucosal injury.^[55]^ Additionally, ETS2 genetic variants may affect T cell differentiation, particularly the balance between Th1, Th17, and regulatory T (Treg) cells, thereby shaping the overall immune landscape in IBD.^[56]^

Future research focusing on functional genomics and transcriptomics is crucial to delineate the specific roles of ETS2 variants in IBD. By integrating genetic, molecular, and clinical data, researchers may identify ETS2-targeted therapeutic approaches that could help modulate immune responses and improve disease outcomes for IBD patients.

## 5. ETS2 in IBD-associated fibrosis and cancer

ETS2 has been increasingly recognized for its role in the development of intestinal fibrosis, a major complication of IBD that leads to stricturing disease and bowel obstruction.^[57]^ Chronic inflammation in IBD drives excessive extracellular matrix deposition, leading to tissue remodeling and fibrosis.^[36]^ ETS2 is involved in key signaling pathways, such as transforming growth factor-beta (TGF-β) and Wnt, which regulate fibroblast activation and EMT. The dysregulation of ETS2 in these pathways contributes to fibroblast proliferation and the excessive deposition of collagen, exacerbating intestinal fibrosis.^[58]^ Moreover, ETS2 influences macrophage polarization, particularly promoting M2 macrophages, which are known to release profibrotic cytokines such as TGF-β and IL-13.^[59]^ This process perpetuates the cycle of inflammation and fibrosis, leading to progressive intestinal strictures that often require surgical intervention in patients with Crohn disease.^[60]^

Beyond fibrosis, ETS2 has also been implicated in the increased risk of colorectal cancer (CRC) among IBD patients. Chronic inflammation is a well-established driver of tumorigenesis in the inflamed gut, and ETS2 plays a crucial role in the regulation of oncogenic pathways.^[61,62]^

Furthermore, ETS2 has been shown to regulate genes involved in cell proliferation, apoptosis resistance, and angiogenesis, all of which are key hallmarks of cancer. Increased ETS2 expression in colonic epithelial cells under chronic inflammatory conditions may promote uncontrolled cell growth and DNA damage, accelerating the progression from dysplasia to malignancy.^[16]^

In addition to its direct effects on epithelial cells, ETS2 plays a role in the interaction between immune cells and the tumor microenvironment.^[63]^ Macrophages and fibroblasts influenced by ETS2 signaling can contribute to tumor progression by secreting cytokines and growth factors that enhance tumor cell survival.^[64]^ Moreover, ETS2 has been linked to resistance mechanisms in cancer therapy, suggesting that its modulation could have therapeutic implications for preventing CRC development in IBD patients.^[65]^ Given its involvement in both fibrotic and neoplastic pathways, targeting ETS2 may provide novel strategies to mitigate the long-term complications of IBD, particularly in individuals at high risk for stricturing disease and malignancy.^[66]^

ETS2 has been increasingly recognized for its role in the development of intestinal fibrosis, a major complication of IBD that leads to stricturing disease and bowel obstruction.^[67]^ Chronic inflammation in IBD drives excessive extracellular matrix deposition, leading to tissue remodeling and fibrosis. ETS2 is involved in key signaling pathways, such as TGF-β and Wnt, which regulate fibroblast activation and EMT. The dysregulation of ETS2 in these pathways contributes to fibroblast proliferation and the excessive deposition of collagen, exacerbating intestinal fibrosis.^[68]^ Moreover, ETS2 influences macrophage polarization, particularly promoting M2 macrophages, which are known to release profibrotic cytokines such as TGF-β and IL-13.^[69]^

This process perpetuates the cycle of inflammation and fibrosis, leading to progressive intestinal strictures that often require surgical intervention in patients with Crohn disease.^[60]^

In addition to its direct effects on epithelial cells, ETS2 plays a role in the interaction between immune cells and the tumor microenvironment. Macrophages and fibroblasts influenced by ETS2 signaling can contribute to tumor progression by secreting cytokines and growth factors that enhance tumor cell survival.^[63]^ Moreover, ETS2 has been linked to resistance mechanisms in cancer therapy, suggesting that its modulation could have therapeutic implications for preventing CRC development in IBD patients.^[24,70,71]^ Given its involvement in both fibrotic and neoplastic pathways, targeting ETS2 may provide novel strategies to mitigate the long-term complications of IBD, particularly in individuals at high risk for stricturing disease and malignancy.^[66]^

## 6. Therapeutic implications of ETS2 in IBD

ETS2 has emerged as a potential biomarker for disease progression in IBD, given its involvement in immune dysregulation, epithelial barrier dysfunction, fibrosis, and cancer development.^[72]^ As a transcription factor that regulates multiple inflammatory pathways, ETS2 expression levels may correlate with disease severity, treatment response, and risk of complications such as fibrosis and colorectal cancer.^[72,73]^ Studies have suggested that increased ETS2 expression in immune and epithelial cells is associated with heightened pro-inflammatory cytokine production, which may serve as an indicator of ongoing mucosal inflammation.^[9,73]^ Additionally, ETS2-driven alterations in macrophage polarization and fibroblast activation could help predict the likelihood of stricturing disease in Crohn disease patients. The ability to measure ETS2 expression through tissue biopsies or noninvasive blood markers may allow for early detection of disease progression and personalized treatment strategies for IBD patients.^[20,74–76]^

Another potential therapeutic avenue is the modulation of ETS2-regulated genes involved in epithelial barrier function. Since ETS2 influences tight junction protein expression and intestinal permeability, targeting downstream pathways may help restore barrier integrity and reduce inflammation.^[77]^ Moreover, given the role of ETS2 in fibrosis and colorectal cancer development, anti-fibrotic agents or epigenetic regulators that modify ETS2 expression could be valuable in preventing long-term complications in IBD patients. Future research focused on understanding the precise molecular mechanisms of ETS2 in IBD could pave the way for novel targeted therapies, offering improved disease control and better clinical outcomes for patients.^[78]^

## 7. Summary of existing literature on ETS2 in inflammatory bowel disease

The role of ETS2 in IBD has been explored across diverse study categories, including experimental models, genetic association studies, and translational research. Mechanistic studies using in vitro cell cultures and animal models have consistently shown that ETS2 regulates epithelial cell homeostasis, including proliferation, apoptosis, and differentiation.^[36–40]^ These studies demonstrated that ETS2 modulates the expression of tight junction proteins (claudin, occludin, zonula occludens-1), which are essential for intestinal barrier integrity.^[42–44]^ Disruption of ETS2, especially under inflammatory cytokine influence (e.g., TNF-α, IFN-γ), leads to tight junction disassembly and increased permeability,^[45,46]^ a hallmark of IBD pathogenesis.

In the domain of genetic studies, GWAS have highlighted ETS2 as a gene of interest due to its location on chromosome 21q22.3 and its role in immune signaling.^[50–52]^ Although not among the top GWAS hits, certain SNPs in or near ETS2 have shown associations with chronic inflammation and epithelial dysfunction. Functional genomics further supports these findings, revealing that ETS2 variants may influence cytokine profiles and immune cell polarization, particularly affecting M1/M2 macrophage balance and T cell responses.^[9,19,53–56]^

Translational and clinical relevance has emerged from studies linking ETS2 to fibrosis and CRC in IBD patients. ETS2 activates TGF-β and Wnt signaling pathways, promoting fibroblast activation, EMT, and extracellular matrix deposition.^[57–60,68,69]^ Moreover, its overexpression in chronically inflamed tissues correlates with tumorigenic processes such as angiogenesis and apoptosis resistance.^[16,24,61–66,70,71]^

Despite promising findings, limitations include reliance on preclinical models, lack of standardized ETS2 expression assays, small sample sizes in human studies, and limited functional validation of SNPs. Future research should focus on multi-omics integration, longitudinal patient cohorts, and targeted ETS2 modulation strategies to fully assess its clinical utility.^[72–78]^

## 8. Conclusion

In conclusion, ETS2 plays a pivotal role in the pathogenesis of IBD through its regulation of immune responses, epithelial barrier function, and fibrosis.

Genetic variants of ETS2 may contribute to disease susceptibility, and its dysregulation is associated with the progression of fibrosis and the increased risk of colorectal cancer in IBD patients. Given its involvement in multiple disease pathways, ETS2 holds promise as a biomarker for disease progression and a potential therapeutic target. Future research into ETS2’s molecular mechanisms will be crucial in developing novel strategies for managing IBD and its complications. Targeting ETS2 may offer a new avenue for improving clinical outcomes and enhancing personalized treatment approaches.

## Author contributions

**Conceptualization:** F.N.U. Aakash, Abida Parveen.

**Data curation:** F.N.U. Aakash.

**Formal analysis:** F.N.U. Aakash, F.N.U. Partab.

**Investigation:** Aasta Kumari.

**Methodology:** F.N.U. Aakash, Aasta Kumari, F.N.U. Shweta, Abida Parveen.

**Project administration:** F.N.U. Shweta, F.N.U. Rumela, F.N.U. Partab.

**Resources:** Sunny Kumar, F.N.U. Shweta, Shivam Singla.

**Supervision:** F.N.U. Aakash, F.N.U. Rumela, Abida Parveen.

**Validation:** F.N.U. Rumela, F.N.U. Partab, Shivam Singla.

**Visualization:** Sunny Kumar, F.N.U. Rumela.

**Writing – original draft:** Sunny Kumar, Aasta Kumari, F.N.U. Shweta, F.N.U. Rumela, F.N.U. Partab, Shivam Singla, Abida Parveen.

**Writing – review & editing:** F.N.U. Aakash, Sunny Kumar, Aasta Kumari, F.N.U. Shweta, F.N.U. Rumela, F.N.U. Partab, Shivam Singla, Abida Parveen.
